# Supplementary material for: Porphyromonas gingivalis Peptidyl Arginine Deiminase (PPAD) in the Context of the Feed-Forward Loop of Inflammation in Periodontitis
Source: Int J Mol Sci. 2023 Aug 18;24(16):12922. doi: 10.3390/ijms241612922 (PMC10454286; doi:10.3390/ijms241612922)
Supplement: Supplementary file 1 [file ijms-24-12922-s001.zip › ijms-2515039-supplementary.pdf]

Supplementary data

Supplementary figures

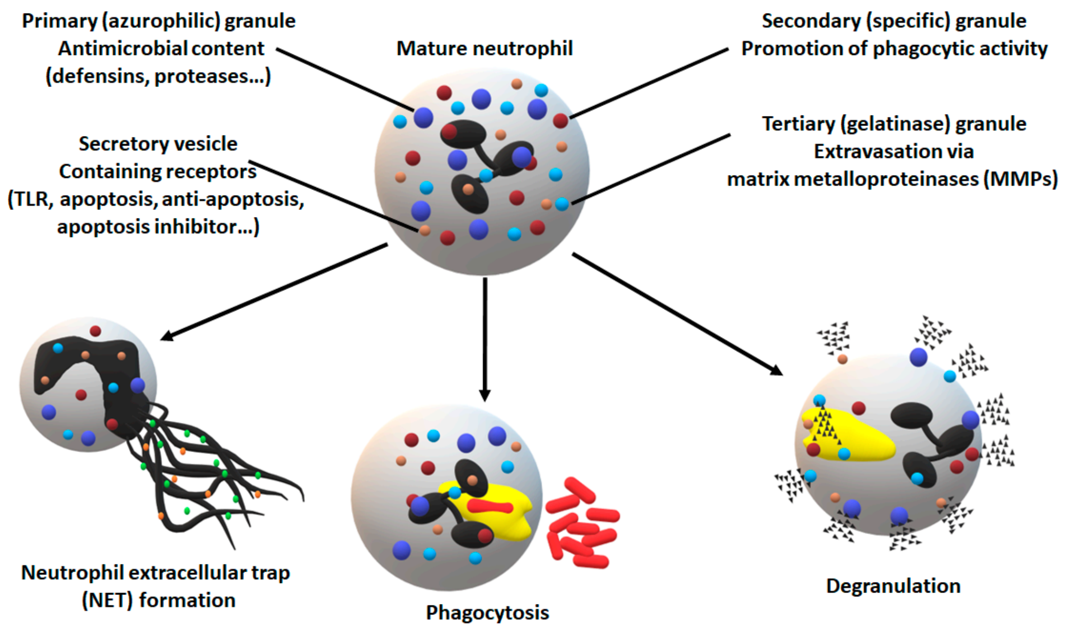

Figure S1. Neutrophil defense mechanisms
Neutrophils are armed with a broad arsenal of strategies not just to eliminate invading pathogens, but also to further activate the host immune system. NET, as its name suggests, is a mechanism to trap and eliminate pathogens similarly to internalization and phagocytosis. Degranulation shows an immense variety in terms of content, destination, and target including not just proteases and degradative enzymes, but also signaling molecules which activate downstream immune responses.

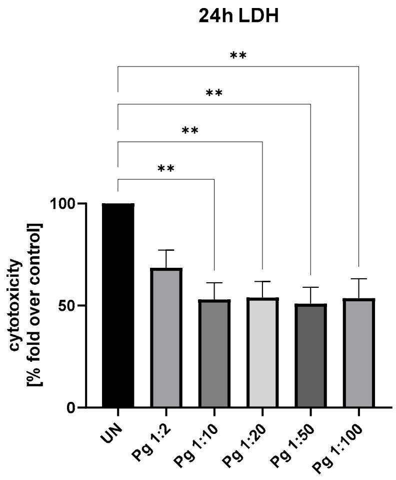

Figure S2. *P. gingivalis* ATCC 33277 does not exhibit cytotoxic effect towards neutrophils. Neutrophils were either left untreated or stimulated for 24 h, with *P. gingivalis* wild-type strains ATCC33277 at the multiplicity of infection (MOI) of 1:2, 1:10, 1:20, 1:50, and 1:100. LDH leakage was measured using the CyQUANT LDH cytotoxicity assay kit according to the manufacturer's protocol. The data was analyzed as a fold over control. Quantification of results from three independent wild-type cell lines; bars show means ± S.E.M. Differences between groups were calculated by One-way ANOVA followed by Brown-Forsythe test; ** $p \leq 0.01$.

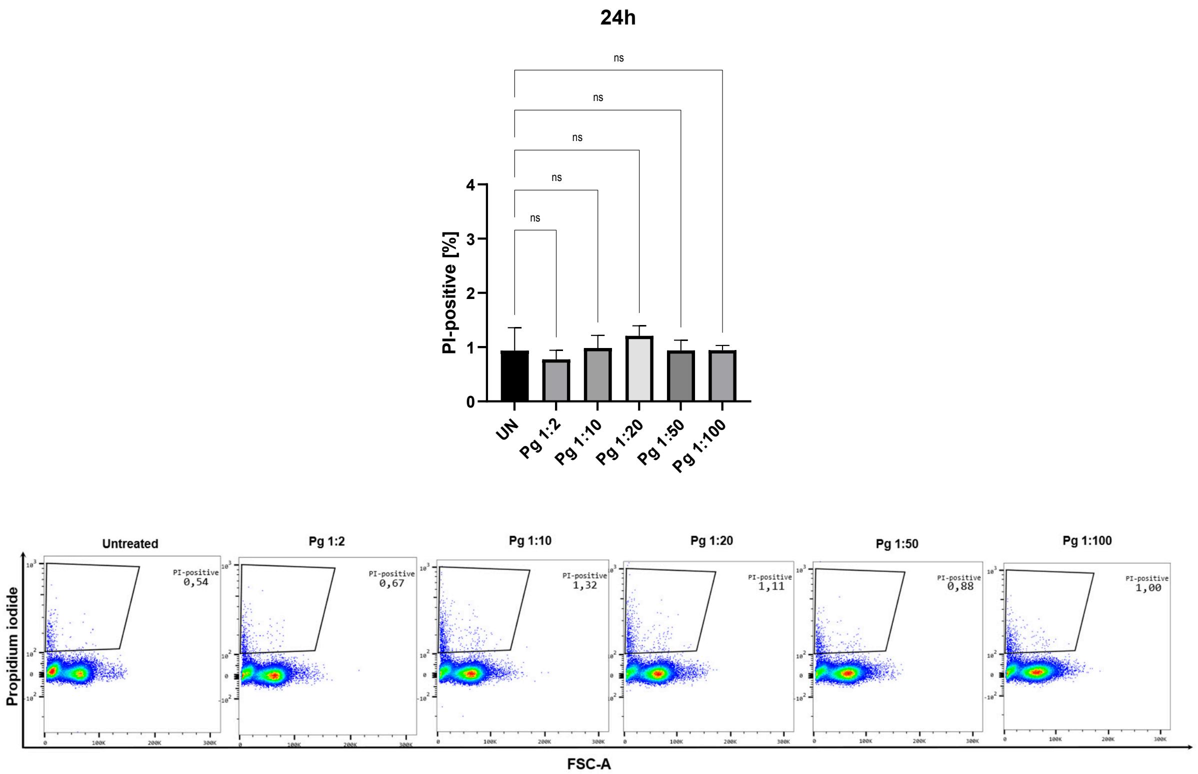

Figure S3. *P. gingivalis* ATCC 33277 did not promote the lytic cell death.
Neutrophils were either left untreated or stimulated for 24 h, with *P. gingivalis* wild-type strains ATCC33277 at the multiplicity of infection (MOI) of 1:2, 1:10, 1:20, 1:50, and 1:100. **(Upper panel)** Lytic cell death was analyzed by flow cytometry by propidium iodide (PI) staining. **(Lower panel)** Representative dot plots showing PI staining analyzed by FACS and FlowJo software. The percentage of propidium iodide-positive cells was gated. Quantification of results from three independent wild-type cell lines; bars show means ± S.E.M. Differences between groups were calculated by One-way ANOVA followed by Brown-Forsythe test; ns – not significant.

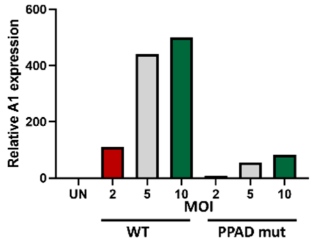

Figure S4. Quantification of the anti-apoptotic protein expression
A1 protein expression levels were calculated from Western Blot images on Figure 2b using ImageJ software, and the ratios were obtained relative to GAPDH and the ratios were obtained relative to the control group (untreated neutrophils challenged with WT-*Pg*). GAPDH was used as an internal control for equal loading. Bar graphs show A1 protein expression relative to GAPDH signal intensity.

Supplementary Materials and Methods

Propidium iodide staining

The percentage of necrotic cells was determined using propidium iodide (BioLegend). Cells were harvested and immediately, before FACS analysis, propidium iodide was added to the FACS tube (1:100). The samples were analyzed by flow cytometry on a BD LSRFortessa™ flow cytometer. FACS data were analyzed with FlowJo Software.

LDH assay

After 24 hours post stimulation, the cells were harvested, centrifuged and the supernatants were collected into 96-well black microplates, and immediately the LDH assay was performed. Levels of released lactate dehydrogenase (LDH) were measured by CyQUANT™ LDH Cytotoxicity Assay – Fluorescence Kit according to the manufacturer's protocols. Fluorescence was measured at an excitation of 560 nm and an emission of 590 nm using Flex Station 3. The results obtained were normalized to the control sample (untreated cells).

Protein quantification

Relative A1 protein expression was calculated based on the signal intensity of A1 protein relative to GAPDH signal intensity for each of the conditions using the Image J and Graph Pad Prism 8 programs.
